# Sonic Hedgehog Signaling in Cranial Neural Crest Cells Regulates Microvascular Morphogenesis in Facial Development

**DOI:** 10.3389/fcell.2020.590539

**Published:** 2020-10-07

**Authors:** Miranda R. Sun, Hannah M. Chung, Veronika Matsuk, Dustin M. Fink, Matthew J. Stebbins, Sean P. Palecek, Eric V. Shusta, Robert J. Lipinski

**Affiliations:** ^1^Department of Comparative Biosciences, School of Veterinary Medicine, University of Wisconsin–Madison, Madison, WI, United States; ^2^Molecular and Environmental Toxicology Center, University of Wisconsin–Madison, Madison, WI, United States; ^3^Department of Chemical and Biological Engineering, University of Wisconsin–Madison, Madison, WI, United States; ^4^Department of Neurological Surgery, University of Wisconsin–Madison, Madison, WI, United States

**Keywords:** perivascular, pericyte, cranial neural crest, Sonic hedgehog, orofacial clefting, microvascular morphogenesis

## Abstract

Sonic hedgehog (Shh) pathway disruption causes craniofacial malformations including orofacial clefts (OFCs) of the lip and palate. In normal craniofacial morphogenesis, Shh signals to multipotent cranial neural crest cells (cNCCs) and was recently discovered to regulate the angiogenic transcriptome, including expression markers of perivascular cells and pericytes. The mural cells of microvasculature, pericytes in the brain and face differentiate from cNCCs, but their role in facial development is not known. Here, we examined microvascular morphogenesis in a mouse model of Shh pathway antagonist-induced cleft lip and the impact of cNCC-specific Shh pathway activation in a cNCC-endothelial cell co-culture system. During cleft pathogenesis *in vivo*, disrupted microvascular morphogenesis localized with attenuated tissue outgrowth in the medial nasal processes that form the upper lip. *In vitro*, we found that human umbilical vein endothelial cell (HUVEC) cord formation was not affected by direct Shh pathway perturbation. However, in a co-culture system in which cNCCs directly interact with endothelial cells, cNCC-autonomous Shh pathway activity significantly prolonged endothelial cord network stability. Taken together, these findings support the premise that Shh pathway activation in cNCCs promotes pericyte-like function and microvascular stability. In addition to suggesting a previously unrecognized role for Shh signaling in facial development, these studies also identify perivascular differentiation and microvascular morphogenesis as new focuses for understanding normal and abnormal craniofacial development.

## Introduction

Orofacial clefts (OFCs) of the lip and palate are among the most common human birth defects and impose a substantial burden on the health and well-being of affected individuals (Wehby and Cassell, 2010; Watkins et al., 2014). OFCs are etiologically complex, and the cellular processes and molecular mechanisms underlying normal and abnormal craniofacial morphogenesis are incompletely understood, hindering the development of effective prevention strategies. Sonic hedgehog (Shh) signaling is required for normal facial morphogenesis and plays a critical role in the outgrowth of the facial processes that form the upper lip and palate (Bush and Jiang, 2012; Kurosaka, 2015). Disruption of the Shh pathway causes OFCs in animal models and is linked to OFC pathogenesis in humans (Roessler et al., 1996, 2003; Heyne et al., 2015a; Yu et al., 2017). However, a cohesive cellular mechanism explaining the role of Shh signaling in normal and abnormal facial development has yet to be established.

Recently, we found that Shh signaling regulates the angiogenic and perivascular transcriptomes in a mouse model of cleft lip (Everson et al., 2018). While Shh signaling has previously been shown to affect angiogenesis and vascular development, the underlying mechanisms are not well defined (Chapouly et al., 2019). Some studies suggest that Shh signaling acts directly on endothelial cells to promote vascular function (Renault et al., 2010; Alvarez et al., 2011). One of these studies has reported canonical pathway activation in endothelial cells after SHH ligand stimulation (Alvarez et al., 2011), but whether Shh signaling promotes angiogenesis and vascular development directly through endothelial cells remains controversial.

Other lines of investigation have linked Shh signaling to pericytes, the mural cells of the microvasculature, which signal to adjacent endothelial cells to promote proliferation, angiogenesis, and microvascular stability (Armulik et al., 2005, 2011; Chapouly et al., 2019). Pericytes have received resurgent interest as they have been identified to play important roles in development, wound healing, tissue regeneration, and disease pathogenesis (Armulik et al., 2011). Shh signaling has been shown to regulate pericyte biology in the liver and may also promote pericyte recruitment in several organs (Armulik et al., 2011; Machado and Diehl, 2018). Craniofacial pericytes are derived from multipotent cranial neural crest cells (cNCCs), which also form most of the cartilage and bone of the head (Etchevers et al., 2001; Jiang et al., 2002; Stebbins et al., 2019). While cNCC-derived pericytes have been less-studied than the mesoderm-derived pericytes of the trunk, their involvement in blood-brain barrier development and maintenance as well as retina development and disease has received substantial recent attention (Trost et al., 2016; Santos et al., 2018; Brown et al., 2019). In contrast, our understanding of the signaling pathways that drive cNCC-to-pericyte differentiation and the role of pericyte-endothelial cell interactions in normal and abnormal craniofacial and upper lip morphogenesis is extremely limited.

In this study, we use *in vivo* and *in vitro* models to investigate the effect of Shh signaling on microvasculature in the developing upper lip. A mouse model of Shh pathway antagonist-induced cleft lip is used to examine microvascular morphogenesis during normal and abnormal upper lip development. We then establish an *in vitro* co-culture model using cNCC-derived pericytes and endothelial cells to dissect the individual cell-type contributions of Shh pathway perturbation on microvascular stability. Our findings suggest a previously unrecognized role for Shh signaling in facial development and identify perivascular differentiation and microvascular morphogenesis as new focuses for understanding normal and abnormal craniofacial development.

## Materials and Methods

### Animal Studies

All studies were conducted in strict accordance with the recommendations in the Guide for the Care and Use of Laboratory Animals of the National Institutes of Health. The protocol was approved by the University of Wisconsin–Madison, School of Veterinary Medicine Institutional Care and Use Committee (Protocol No. 13–081.0). Male and female C57BL/6J mice (*Mus musculus*, Jackson Laboratory strain 00664) were housed under specific-pathogen-free conditions in disposable, ventilated cages (Innovive). Rooms were maintained at 22 ± 2°C and 30–70% humidity with a 12-h light, 12-h dark cycle. Mice were fed Irradiated Soy Protein-Free Extruded Rodent Diet (Catalog No. 2920x; Envigo Teklad Global) until day of copulation plug when dams were switched to Irradiated Teklad Global 19% Protein Extruded Rodent Diet (Catalog No. 2919; Envigo Teklad Global).

One to two nulliparous female mice were placed with a single male for 1–2 h and subsequently examined for the presence of copulation plugs. The beginning of the mating period was designated as gestational day (GD)0. Pregnancy was confirmed by assessing weight gain at GD7, as previously described (Heyne et al., 2015b). Pregnant dams were administered 90 or 120 mg/kg/day cyclopamine (LC Laboratories, CAS #4449-51-8) or vehicle alone from GD8.25 to approximately GD9.375 by subcutaneous infusion exposure using ALZET 2001D micro-osmotic pumps (Cupertino, CA, United States) as previously described (Lipinski et al., 2008, 2010; Everson et al., 2017; Ansen-Wilson et al., 2018). Pregnant dams were euthanized by carbon dioxide inhalation followed by cervical dislocation for embryo collection at GD9.25 or GD11 ± 1 h.

### Light Imaging

GD11 embryos for light imaging were fixed in Bouin’s solution, and representative images were taken using a MicroPublisher 5.0 camera (QImaging) mounted on an Olympus SZX-10 stereomicroscope. A single litter each of vehicle- and 120 mg/kg/day cyclopamine-exposed embryos was collected for imaging.

### PECAM1 Whole-Mount Immunohistochemistry for Quantitative Analysis

GD11 embryos exposed to vehicle or 90 mg/kg/day cyclopamine were fixed in 4% paraformaldehyde (PFA) in PBS for 6 h before dehydration through a graded series into 100% methanol for storage at −20°C. After rehydration, embryos were hemisected, and one half of each embryo was embedded in 4% agarose in PBS. A vibrating microtome was used to make a single 400-μm section of the lambdoidal junction, the rostral-most aspect of the developing facial processes containing the medial nasal, lateral nasal, and maxillary processes (MNP, LNP, and MxP, respectively). Sections were blocked in PBS with 0.3% Triton X, 0.2% BSA, and 5% goat serum for 1 h at room temperature followed by incubation with an antibody against PECAM1 (1:200 dilution, BD Pharmingen 557355) overnight at 4°C. A goat anti-rat secondary antibody conjugated with Alexa Fluor 488 (1:200 dilution, Jackson ImmunoResearch 112-546-003) and 60 nM DAPI were incubated with the sections at room temperature for 2 h. Sections were mounted on microscope slides with Vectashield mounting medium (Vector Laboratories) and imaged using a Leica SP8 confocal microscope. Four vehicle-treated litters and four cyclopamine-treated litters were collected, and 1–4 embryos from each litter were selected for analysis based on phenotype (cleft and non-cleft in the cyclopamine group) and proper staging (total *n* = 10 vehicle-exposed embryos, *n* = 12 cyclopamine-exposed embryos).

### Image Processing and Microvasculature Analysis

Z-stacks from confocal imaging of PECAM1-stained lambdoidal junction sections were imported into ImageJ, and image stacks for all samples were three-dimensionally rotated for consistent orientation. A maximum projection of the rostral-most 50 μm of each re-oriented stack was used for subsequent analysis. A standard-size square region of interest (ROI) was drawn within the vascularized mesenchymal tissue of the MNP and analyzed using AngioTool software. Lambdoidal sections from embryos exposed to 90 mg/kg/day cyclopamine were classified as having either a non-clefted (NC) or cleft lip (CL) phenotype. Samples were considered NC if the MNP was touching or fused with the MxP, and samples were considered CL if the MNP was not touching the MxP. Analysis was carried out by a single investigator blinded to sample treatment group.

### PECAM1 and Laminin Immunohistochemistry

For qualitative *in vivo* PECAM1 and Laminin co-immunostaining, a PFA-fixed control GD11 embryo was coronally sectioned into 50-μm sections using a vibrating microtome and subsequently immunostained following the above protocol for whole-mount immunohistochemistry. Primary antibodies against PECAM1 (1:200 dilution, BD Pharmingen 557355) and Laminin (1:1000 dilution, Sigma-Aldrich L9393) were used to stain for microvasculature. A goat anti-rat secondary antibody conjugated with Alexa Fluor 488 (1:200 dilution, Jackson ImmunoResearch 112-546-003) and a goat anti-rabbit secondary antibody conjugated with DyLight 594 (1:200 dilution, Thermo Scientific 35560) were used. Sections were imaged on a Keyence BZ-X700 fluorescent microscope.

### Cell Culture

Human umbilical vein endothelial cells (HUVECs) were purchased from Lonza, cultured in EGM-2 according to manufacturer recommendations, and used for experiments at passage 5. O9-1 cells, a mouse cNCC line, were cultured as described previously (Ishii et al., 2012). O9-1 cNCCs stably expressing a mutated constitutively activated form of Smoothened (SMO^M 2^) along with GFP (cNCC-SMO), or GFP alone as a control (cNCC-GFP), were generated as previously described (Everson et al., 2017). To differentiate O9-1 cNCCs into pericytes, cells were cultured in E6 media with 10% FBS, following a previously described protocol (Stebbins et al., 2019). Pericyte-differentiated cNCC-GFP and cNCC-SMO were designated as cNCC-GFP-PC and cNCC-SMO-PC, maintained in E6 media with 10% FBS, and used for experiments at passages 8–11. Five biological replicates of cNCC-GFP-PC and cNCC-SMO-PC were collected for gene expression analyses.

For SHH ligand treatment, parent O9-1 cNCCs were plated at 5 × 10^5^ cells/ml (0.4 ml per well in a 24-well plate) and allowed to attach in complete medium for 24 h before media were replaced with DMEM containing 1% FBS and recombinant human SHH ligand (0.4 μg/ml final concentration, R&D Systems 1845-SH), made in a stock solution at 100 μg/ml in sterile-filtered 5 mg/ml bovine serum albumin (BSA) in PBS, or equivalent volume of BSA vehicle. After 48 h, cells were harvested for RNA isolation. Five biological replicates were collected for gene expression analyses.

### Matrigel Cord Formation Assay

For cord formation assays, 100 μl of Matrigel (Corning) was added to the wells of a chilled 48-well plate and incubated at 37°C for at least 30 min according to manufacturer recommendations. In the HUVEC-alone experiments, cells were trypsinized, and 1.58 × 10^4^ HUVECs were resuspended in 300 μl EGM-2 per well with 0.4 μg/ml SHH ligand or BSA vehicle and 200 nM vismodegib (dissolved in 100% DMSO) or DMSO vehicle and plated into wells containing Matrigel. A total of 12 biological replicates per treatment group were plated for phase contrast imaging. For RNA extraction, four wells were pooled for each experimental group 12 h after plating, and a total of five biological replicates were plated.

For the cNCC-endothelial cell co-culture experiments, cells were trypsinized, and 1.4 × 10^4^ HUVECs and 0.18 × 10^4^ cNCC-GFP-PC or cNCC-SMO-PC (8:1 ratio, based on not shown preliminary optimization) were resuspended in 300 μl EGM-2 per well and plated into wells containing Matrigel. Pericyte-differentiated cNCCs were switched to EGM-2 media 24 h before plating. A total of 12 biological replicates per treatment group were plated for phase contrast imaging (one well had a tear in the Matrigel within the imaging field; therefore, that replicate was excluded, and a total of 11 biological replicates were used for analysis).

### Matrigel Cord Formation Assay Analysis

Phase contrast images were taken with a MicroPublisher 5.0 camera (QImaging) mounted on a Nikon Eclipse TS100 with a 4x objective at 12 and 22 h after cells were plated for cord formation assays. Images were imported into ImageJ and analyzed using the Angiogenesis plugin. ImageJ was used to modify cord network schematics generated by the Angiogenesis plugin for better visibility in representative images.

### Immunocytochemistry and Imaging

At 6–8 h after plating, cells in cord formation assays were fixed in 4% PFA in PBS for 15 min at room temperature. Cords were blocked in PBS with 0.3% Triton X, 0.2% BSA, and 5% goat serum for 1 h at room temperature and then incubated overnight at 4°C with primary antibodies against PECAM1 (1:200 dilution, Proteintech 66065-1-Ig) or Laminin (1:1000 dilution, Sigma-Aldrich L9393). Cords were incubated with a goat anti-rabbit or anti-mouse secondary antibody conjugated to DyLight 594 (1:200, Thermo Scientific 35560 and 35511) and 60 nM DAPI for 1 h at room temperature for detection. A Keyence BZ-X700 was used for fluorescent imaging.

### RNA Extraction, Reverse Transcription, and qPCR

A GE Illustra RNA spin mini kit was used for RNA extraction according to manufacturer protocols. A total of 100–500 ng of RNA was used for cDNA synthesis using GoScript reverse transcription reaction kits (Promega). Singleplex quantitative real-time PCR (qPCR) was performed on a Bio-Rad CFX96 system using SsoFast EvaGreen Supermix (Bio-Rad), and primers were designed using the IDT PrimerQuest tool. qPCR primer sequences are shown in Table 1. Target gene and species specificity was confirmed using NCBI Primer-BLAST. Mouse *Gapdh* or human *GAPDH* was used as the housekeeping gene, and analyses were conducted using the 2^–ΔΔ*Ct*^ method.

**TABLE 1:** Primer sequences used for qPCR.

Primer	Species	Sequence
GAPDH forward	human	CCACATCGCTCAGACACCAT
GAPDH reverse	human	GCAACAATATCCACTTACCAGAGTTAA
GLI1 forward	human	AATGCTGCCATGGATGCTAGA
GLI1 reverse	human	GAGTATCAGTAGGTGGGAAGTCCATAT
PTCH1 forward	human	CGCTGGGACTGCTCCAAGT
PTCH1 reverse	human	GAGTTGTTGCAGCGTTAAAGGAA
Angpt1 forward	mouse	CCATTCCAGCAGCAAGTGGTTA
Angpt1 reverse	mouse	TGAAGTGCAGCAAGCTGAGAAG
Angpt2 forward	mouse	CCACATTCCTCTAAGCACGGTTTC
Angpt2 reverse	mouse	TAACCTGTGCCCACCACTTAGA
Cd248 forward	mouse	GTTGCTGGATGATGGAGAAGAAGG
Cd248 reverse	mouse	GCCAAAGTCAGGTGGATGTGTAG
Cspg4 forward	mouse	CAAATGCTCCCGTCCTCACTAAC
Cspg4 reverse	mouse	AGGCTGCTCCATCACCTCATA
Edn1 forward	mouse	CCAAGCGCTGTTCCTGTTCTT
Edn1 reverse	mouse	TGGAAGAACCTCCCAGTCCATAC
Foxc1 forward	mouse	TTCTTGCGTTCAGAGACTCG
Foxc1 reverse	mouse	TCTTACAGGTGAGAGGCAAGG
Foxd1 forward	mouse	CGTTTCTAGATTCTCACTCCTC
Foxd1 reverse	mouse	TCCACTGTGGTCCCTTTA
Foxf2 forward	mouse	TTCTCTAGTTCCCTGGCTCAGTAG
Foxf2 reverse	mouse	TGTTCTTTGGCACCTGTATCCG
Gapdh forward	mouse	AGCCTCGTCCCGTAGACAAAAT
Gapdh reverse	mouse	CCGTGAGTGGAGTCATACTGGA
Gli1 forward	mouse	GGAAGTCCTATTCACGCCTTGA
Gli1 reverse	mouse	CAACCTTCTTGCTCACACATGTAAG
Lama2 forward	mouse	CGCACCTTGAATGCAGACTTGA
Lama2 reverse	mouse	GCACATCCCGCCAACTGAAATA
Ptch1 forward	mouse	CTCTGGAGCAGATTTCCAAGG
Ptch1 reverse	mouse	TGCCGCAGTTCTTTTGAATG
Tbx18 forward	mouse	ACGAAATAGGCACCGAGATG
Tbx18 reverse	mouse	ATTGCTGGTGAGGGTCTAATC

### Statistics

Graphpad Prism 8 was used for all statistical analysis. Results from *in vivo* experiments were analyzed using unpaired statistical tests. Two-tailed *t*-tests were used to analyze the differences between proximal and distal microvasculature. Ordinary one-way analysis of variance (ANOVA) with Tukey’s *post hoc* test for multiple comparisons was used for analyses of microvasculature between vehicle and cyclopamine-exposed groups. *In vitro* experiments were run in pairwise manner and paired statistical tests were used. Ordinary one-way ANOVA with Tukey’s *post hoc* test for multiple comparisons was used for cord formation assays and Shh pathway gene expression in HUVEC-alone experiments. Two-tailed *t*-tests were used to analyze differences in gene expression in parent cNCCs and pericyte-differentiated cNCCs as well as in co-culture cord formation between groups. An alpha value of 0.05 was maintained for determination of significance for all experiments.

## Results

### Microvascular Density Is Increased at the Distal Tip of the Medial Nasal Processes

**FIGURE 1:**
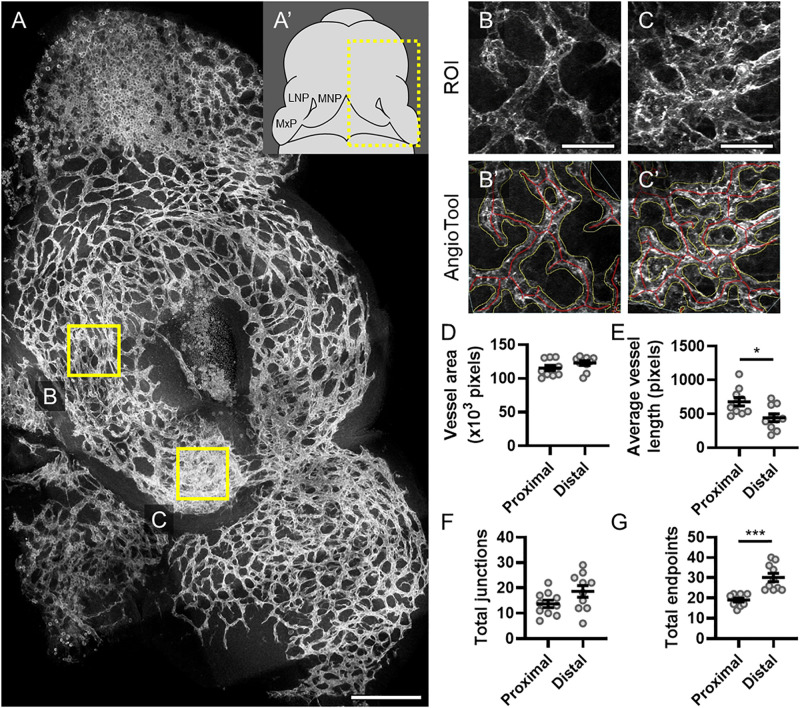
Microvascular density is increased in the distal medial nasal process. **(A)** A representative maximum projection of a 180-μm z-stack of the lambdoidal junction stained for PECAM1 of a vehicle-exposed GD11 embryo is shown with the proximal **(B)** and distal **(C)** regions of the medial nasal process (MNP) indicated. Scale bar is 200 μm. **(A’)** Schematic of a GD11 embryo head with the MNP, lateral nasal process (LNP), and maxillary process (MxP) labeled. Representative regions of interest (ROIs) of the microvasculature of the proximal **(B)** and distal **(C)** MNP from a maximum projection of the rostral-most 50 μm of each section are shown. Scale bars are 50 μm. The vascular networks identified by AngioTool are shown in **(B’)** and **(C’)**. Vessel areas are outlined in yellow and the vessels lengths are in red. **(D–G)** The vessel area, average vessel lengths, number of junctions, and number of endpoints in the microvasculature from 10 vehicle-exposed embryos were analyzed by AngioTool. Measurements for individual embryos are indicated by the icons, and the means ± SEM are shown. *p < 0.05 and ***p < 0.001 between the indicated groups.

The midface forms through precisely coordinated growth and fusion of three bilaterally paired facial growth centers, the medial nasal, lateral nasal, and maxillary processes (MNP, LNP, and MxP, respectively). We visualized vasculature in these growth centers when the MNP and MxP begin to fuse bilaterally to close the upper lip at gestational day (GD)11.0 in the mouse. Staining for the endothelial marker PECAM1 revealed a network of microvasculature with a dense plexus apparent in the distal aspect of the MNP that extends to make contact and subsequently fuse with the MxP (Figures 1A,A’). AngioTool software was then used to quantitatively compare microvascular characteristics between selected regions of interest (ROIs) representing proximal (Figures 1B,B’) and distal (Figures 1C,C’) regions of the MNP. Vessel area and number of junctions were not significantly different between the proximal and distal regions, but the average vessel length was significantly decreased and the number of endpoints was significantly increased in the distal region, suggesting an increase in the number and therefore density of vessels at the distal tip of the MNP (Figures 1D–G).

### Microvascular Patterning Is Disrupted in Cleft Lip Pathogenesis

**FIGURE 2:**
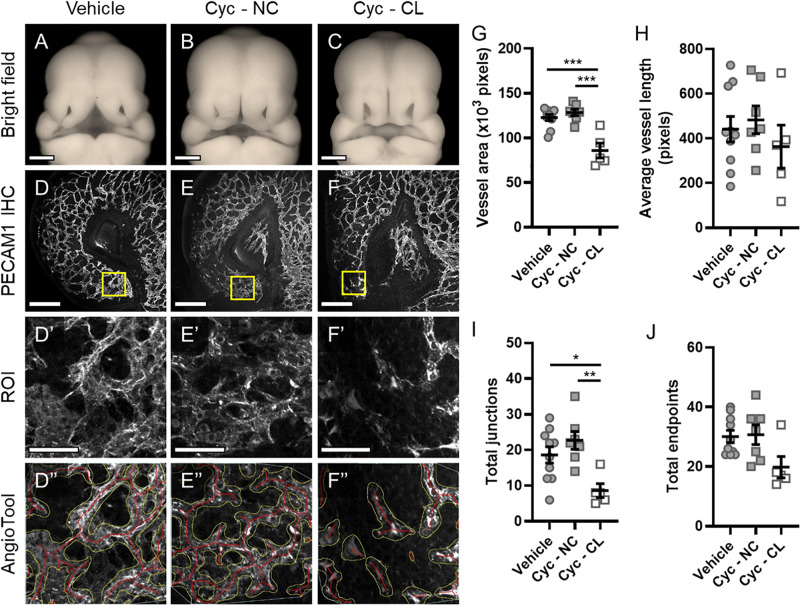
Microvascular patterning is disrupted in cyclopamine-exposed embryos with cleft lip phenotype. **(A–C)** Representative phenotypes in GD11 embryos transiently exposed to vehicle and cyclopamine (cyc) and fixed in Bouin’s solution were imaged. Cyclopamine-exposed embryos were classified as having a non-clefted (NC) or cleft lip (CL) phenotype for analysis. Scale bars are 500 μm. **(D–F)** One lambdoidal junction of each vehicle- and cyclopamine-exposed embryo fixed in 4% PFA was sectioned by vibrating microtome at 400-μm thickness and stained for PECAM1 by whole-mount immunohistochemistry (IHC) to visualize the microvasculature. Maximum projections of the rostral-most 50 μm of the resulting 3D confocal images are shown. Scale bars are 200 μm. **(D’–F’)** Regions of interest (ROIs) were defined at the distal end of the medial nasal process (MNP) as indicated by the boxes in **(D–F)**. Scale bars are 50 μm. **(D”–F”)** An overlay of vessels identified by AngioTool is shown for each ROI in **(D’–F’)**. The vessel areas are outlined in yellow and the vessels lengths are in red. Images are representative of 5–10 embryos per analysis group. **(G–J)** Vessel area, average vessel length, total junctions, and total endpoints in the ROIs were measured by AngioTool. Measurements for individual embryos are indicated by the icons, and the means ± SEM for each group are shown. *p < 0.05, **p < 0.01, and ***p < 0.001 between indicated treatment groups.

Next, we tested whether microvascular morphogenesis was altered in animals with cleft lip using a well-characterized model of Shh pathway inhibition via targeted maternal exposure to the Smoothened antagonist cyclopamine (Lipinski et al., 2008; Heyne et al., 2015a). Smoothened is a G protein-coupled receptor that is necessary for Shh signaling transduction. As previously described, cyclopamine exposure resulted in animals with cleft lip (CL) and without cleft lip (no cleft; NC), which were compared against vehicle-treated control embryos (Figures 2A–C) using the same approach described for Figure 1 (Figures 2D–F”). In the proximal region of the MNP, no significant differences were observed between the vehicle- or cyclopamine-exposed groups in all measured parameters (Supplementary Figure 1). In the distal MNP, however, vessel area and number of junctions were significantly lower in cyclopamine-exposed embryos with cleft lip compared to control embryos or cyclopamine-exposed embryos without clefts (Figures 2G,I). No significant differences were observed between vehicle controls and cyclopamine-exposed embryos without clefts in any measured parameter, and no significant differences in average vessel length or total endpoints were detected between any of the groups (Figures 2G–J).

### Shh Pathway Modulation in Endothelial Cells Does Not Affect Cord Formation

**FIGURE 3:**
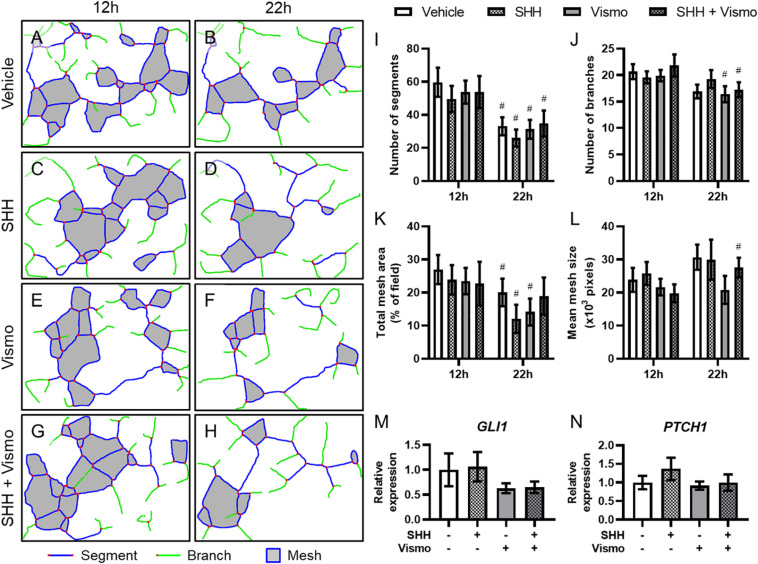
Shh pathway perturbation in endothelial cells does not affect cord formation or downstream Shh pathway targets. **(A–H)** Cord network schematics of HUVECs ± 0.4 μg/ml SHH ligand ± 200 nM vismodegib (vismo) plated on Matrigel were generated from images taken at 12 and 22 h. Blue lines are segments, green lines are branches, red dots are junctions, and filled gray areas are meshes. Images are representative of 12 biological replicates per treatment group. **(I–L)** Number of segments, number of branches, total mesh area, and mean mesh size as measured by the Angiogenesis plugin for ImageJ are shown for the indicated groups at 12 and 22 h. ^#^*p* < 0.05 of the indicated treatment group compared to the same group at 12 h. **(M,N)** Canonical Shh pathway targets *GLI1* and *PTCH1* were assayed for gene expression by qPCR in HUVEC cords treated with SHH and/or vismodegib and harvested at 12 h. Five biological replicates were harvested for gene expression.

To interrogate the mechanism by which Shh signaling regulates microvascular morphogenesis, we cultured HUVECs, which form networks of endothelial cords *in vitro*. HUVECs plated on Matrigel were treated with SHH ligand and/or the potent Smoothened inhibitor vismodegib, and cord networks imaged at 12 and 22 h (Supplementary Figure 2) were analyzed using the Angiogenesis plugin for ImageJ (Figures 3A–H). While there were significant differences within treatment groups from 12 to 22 h, no treatment-dependent differences were detected at either time point (Figures 3I–L). qPCR analysis of a parallel cohort demonstrated no treatment-dependent differences in expression of the conserved Shh pathway target genes *GLI1* and *PTCH1* (Figures 3M,N).

### cNCC-Derived Pericytes Interact With Endothelial Cell Cords

**FIGURE 4:**
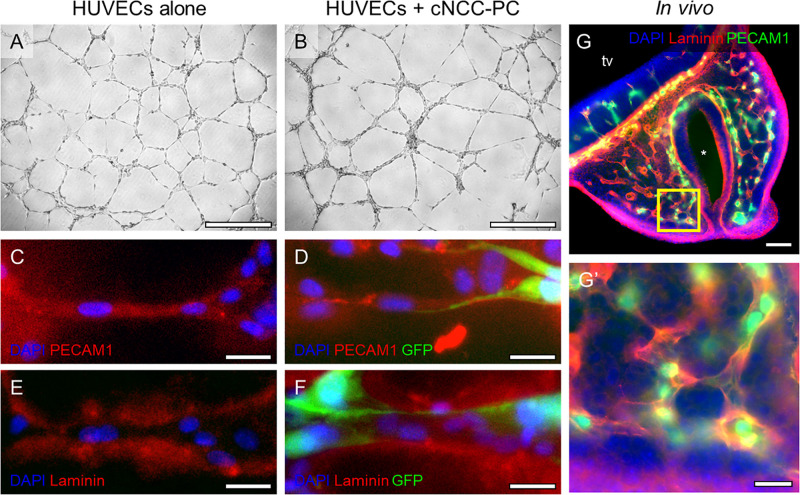
cNCC-derived pericytes interact with endothelial cell cords. **(A,B)** Phase contrast images of HUVECs alone or HUVECs with GFP-expressing pericyte-differentiated cNCCs (cNCC-PC) plated at a ratio of 8:1 on Matrigel for 8 h. Scale bar is 500 μm. **(C–F)** Cords were stained for PECAM1 to mark endothelial cells or for the extracellular matrix protein Laminin. GFP marks cNCC-PC, and DAPI was used to stain nuclei. Scale bars are 25 μm. **(G)** A 50-μm coronal section from a control GD11 embryo head was stained for PECAM1 and Laminin to visualize the microvasculature. tv, telencephalic vesicle, * indicates the nasal pit. Scale bar is 100 μm. **(G’)** A higher magnification image of the field indicated by the box in the medial nasal process in **(G)**. Scale bar is 25 μm.

While Shh pathway modulation did not impact endothelial cord formation directly, we previously observed that Shh signaling altered the angiogenic transcriptome in cultured multipotent cNCCs (Everson et al., 2018). Among the derivatives of cNCCs are pericytes, the mural cells of microvessels that actively signal with endothelial cells. We therefore sought to establish an *in vitro* culture in which cNCC-derived pericytes interact with HUVEC cords in a manner that approximates the *in vivo* pericyte-endothelial relationship. cNCCs were cultured according to a recently described protocol for differentiation of pericytes from human pluripotent stem cell-derived cNCCs (Stebbins et al., 2019), which were then cultured with HUVECs at a 1:8 ratio on Matrigel. By 8 h, cNCC-HUVEC co-cultures formed networks similar to HUVECs alone but with apparently thicker cords (Figures 4A,B). Examination by fluorescent microscopy revealed that GFP-expressing pericyte-differentiated cNCCs approximate and appear to wrap around PECAM1-expressing HUVEC cords (Figures 4C,D). The extracellular matrix protein family of Laminins, an integral component of the basement membrane of vasculature, was found to surround cords formed by HUVECs alone and cNCC-HUVEC co-cultures (Figures 4E,F), resembling the Laminin staining observed in the microvasculature of the MNP *in vivo* (Figures 4G,G’).

### cNCC-Autonomous Shh Pathway Activation Increases Pericyte-Associated Gene Expression

**FIGURE 5:**
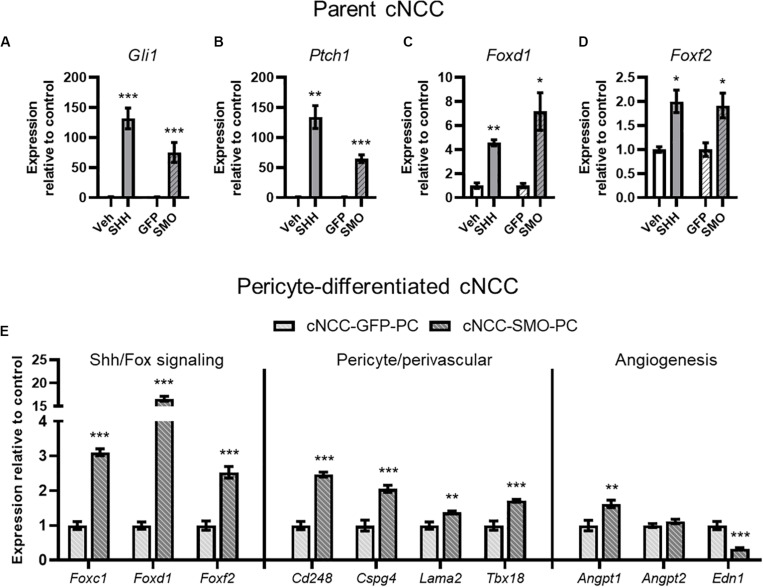
cNCC-autonomous Shh pathway activation increases pericyte-associated gene expression. **(A–D)** SHH ligand treatment or expression of the constitutively active SMO^M 2^ (SMO) induces expression of Shh target genes *Gli1* and *Ptch1* in addition to Shh pathway-regulated Forkhead box transcription factor (Fox) genes. **(E)** Shh/Fox genes, pericyte/perivascular genes, and angiogenic genes are regulated by cNCC-autonomous Shh pathway activation in pericyte-differentiated cNCCs. Five biological replicates were analyzed for all groups. *p < 0.05, **p < 0.01, and ***p < 0.001 compared to respective control group.

Having established an *in vitro* model of direct cNCC-pericyte/HUVEC interaction, we next sought to test the impact of cNCC-specific Shh pathway activation. From parent cNCCs, we generated lines expressing a mutated constitutively active form of Smoothened (SMO^M 2^) and GFP, or GFP alone for control. qPCR analysis showed increased expression of Shh target genes *Gli1* and *Ptch1* in SMO^M 2^-expressing cNCCs that approximated the level of induction achieved by treating parent cells with SHH ligand (Figures 5A,B). Both SHH ligand stimulation and SMO^M 2^ expression also resulted in increased expression of *Foxd1* and *Foxf2* (Figures 5C,D), which have been demonstrated to be Shh target genes and required for brain or kidney pericyte differentiation and function (Jeong et al., 2004; Gomez and Duffield, 2014; Reyahi et al., 2015; Everson et al., 2017, 2018; Fink et al., 2018). We next assessed pericyte-differentiated GFP- or SMO^M 2^-expressing cNCCs by qPCR. Relative to GFP controls, SMO^M 2^-expressing cNCCs exhibited increased expression of multiple pericyte-associated genes, including *Foxc1*, *Foxd1*, *Foxf2*, *Cd248*, *Cspg4*, *Lama2*, and *Tbx18.* SMO^M 2^-expressing pericyte-differentiated cNCCs also exhibited increased expression of pro-angiogenic factor *Angpt1* and decreased expression of anti-angiogenic factor *Edn1* (Figure 5E).

### cNCC-Autonomous Shh Pathway Activation Promotes Cord Stability

**FIGURE 6:**
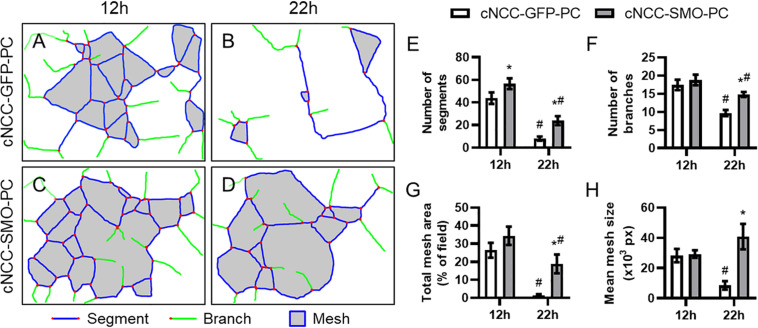
Cell-autonomous Shh pathway activation in cNCC-derived pericytes promotes endothelial cord stability. **(A–D)** Cord network schematics of HUVEC and control cNCC-GFP-PC or Shh pathway-activated cNCC-SMO-PC co-cultures were generated from images taken at 12 and 22 h. Blue lines are segments, green lines are branches, red dots are junctions, and filled gray areas are meshes. Images are representative of 11 biological replicates per group. **(E–H)** Number of segments, number of branches, total mesh area, and mean mesh size were measured by the Angiogenesis plugin for ImageJ for the indicated groups at 12 and 22 h. *p < 0.05 between co-cultures at the same time point, ^#^*p* < 0.05 of the indicated co-culture at 22 h compared to the same co-culture at 12 h.

We next examined whether pericyte-differentiated cNCC-autonomous Shh pathway activation impacts endothelial cord formation or stability. HUVECs were co-cultured on Matrigel with control cNCC-GFP-PC or Shh pathway-activated cNCC-SMO-PC and imaged at 12 and 22 h (Supplementary Figure 3). At 12 h, there was a significant difference in only the number of segments between the two co-cultures (Figure 6E). By 22 h, however, the cord networks in the control co-culture had begun to dissociate while the cord networks in Shh-pathway-activated co-culture remained mostly intact (Figures 6A–D). At 22 h, the number of segments, number of branches, total mesh area, and mean mesh size were significantly greater in co-cultures with cNCC-SMO-PC compared to co-cultures with cNCC-GFP-PC (Figures 6E–H).

## Discussion

Attenuated outgrowth of the midfacial primordia has long been recognized as a morphological mechanism of OFCs. However, relatively little attention has been given to understanding the role of angiogenesis and microvascular biology in facial morphogenesis and cleft pathogenesis. Here, we present evidence suggesting that Shh signaling promotes pericyte-like function in cNCCs and that this activity is necessary for microvascular stability and proper facial morphogenesis. We found that outgrowth of the MNPs is accompanied by dense microvasculature and that both microvascular patterning and outgrowth are disrupted by Shh pathway inhibition in a mouse model of cleft lip. In an *in vitro* co-culture model recapitulating direct interaction of cNCC-derived pericytes and endothelial cells, we found that cNCC-autonomous Shh pathway activation was sufficient to promote cord stability. Together, these findings suggest a previously unrecognized role for Shh signaling in facial development and highlight pericyte-endothelial signaling and microvascular biology as new areas of investigation in OFC etiology and pathogenesis.

Disruption of angiogenesis, which is required for tissue outgrowth by providing oxygen and nutrients, has previously been linked to birth defect pathogenesis. For example, thalidomide-induced limb truncations have been reported to result from direct disruption of angiogenesis, where the perturbation of microvascular morphogenesis was found to precede changes in tissue patterning, cell proliferation, and cell death, during proximal-distal limb outgrowth (Therapontos et al., 2009). More recently, conditional deletion of *Vegfa* in cNCCs was shown to cause cleft palate in the mouse, supporting the premise that disrupted angiogenesis can specifically cause OFCs (Hill et al., 2015). In addition, angiogenic gene expression has been found to be altered in animal models and in patients with OFCs (François-Fiquet et al., 2014; Francois et al., 2017; Everson et al., 2018). The high frequency of cardiovascular abnormalities in patients with OFCs may also support the relevance of angiogenic and vascular dysfunction as an underlying mechanism (Milerad et al., 1997; Sekhon et al., 2011).

During upper lip morphogenesis, we identified a dense microvascular plexus at the distal end of the MNP, which normally extends to make contact and fuse with the MxP to close the upper lip (Figure 1). Using an inducible lineage tracing model, we found previously that SHH-responsive cells during the critical period for cleft lip (GD8.75) populate this distal aspect of the MNP (Supplementary Figure 4; Ansen-Wilson et al., 2018). Here, we show that the microvasculature in this region of the MNP is also disrupted after Shh pathway inhibition (Figure 2). This effect on the microvasculature appeared specific to the distal aspect of the MNP and exclusive to animals with cleft lip resulting from attenuated outgrowth of the MNP. These observations link Shh signaling to both microvascular morphogenesis and tissue outgrowth during upper lip development. Notably, disrupted microvasculature and tissue deficiency identified at GD11 follow Shh pathway inhibition and disruption of the angiogenic transcriptome previously identified at GD9.25 (Everson et al., 2018). However, elucidating whether microvascular defects are the primary cause of the Shh-mediated craniofacial malformation will require further investigation.

The Shh pathway is known to regulate vasculogenesis, angiogenesis, and vascular stability in various aspects of development, wound healing, and tumor formation (Chapouly et al., 2019). During facial development, we propose that, rather than acting directly on endothelial cells, Shh signals through cNCC-derived pericytes, which interact with endothelial cells and maintain microvascular stability. Our results indicate that HUVECs, a cell type commonly used to model endothelial cell function, do not respond directly to Shh pathway modulation. However, cNCC-autonomous Shh pathway activation led to increased expression of pericyte-associated genes and other pro-angiogenic transcriptional responses (Figure 5) and significantly delayed the degeneration of microvessel-like cords in culture (Figure 6).

Pericytes have been increasingly recognized to play a critical role in regulating endothelial cell biology as well as microvascular morphology and homeostasis (Armulik et al., 2011). Lineage tracing studies have shown that, while trunk pericytes are mesoderm-derived, pericyte populations in the head are derived from cNCCs (Etchevers et al., 2001). Although cNCC-derived pericytes of the blood-brain barrier and retina have received substantial research attention, little is known about the molecular drivers of their differentiation in the facial primordia and the role that pericytes play in facial morphogenesis. Expression of the Forkhead box transcription factor (Fox) family genes *Foxc1* and *Foxf2* has been found in brain pericytes and has been shown to be critical for pericyte differentiation and function (Siegenthaler et al., 2013; Reyahi et al., 2015), while lineage tracing has found that *Foxd1*-expressing cells give rise to pericytes in the developing kidney (Gomez and Duffield, 2014). In craniofacial morphogenesis, SHH peptide secreted from the facial ectoderm produces a gradient of target gene expression, including *Foxd1* and *Foxf2*, in the cNCC mesenchyme, with strong and apparent ubiquitous expression nearest the ectoderm (Everson et al., 2017; Fink et al., 2018). Here, we found that Shh pathway activation in cNCCs leads to increased expression of *Foxc1*, *Foxd1*, and *Foxf2* and promotion of pericyte-like activity. Activation of Fox transcription factors in cNCCs may therefore be common drivers of pericyte differentiation and/or function, though it is likely that they act in concert with additional factors secreted by endothelial cells – a premise supported by recent findings that signaling from the endothelium also drive pericyte differentiation (Brandt et al., 2019; Niimi et al., 2019).

Our limited knowledge of pericyte-endothelial biology in facial development stems, at least in part, from paucity of reliable pericyte markers. Pericytes and pericyte-like cells have been reported to express numerous gene products that are classically associated with vascular homeostasis as well as other genes that may serve as important endothelial-pericyte signals including *Pdgfr*β, *Cspg4, Tbx18, Rgs5, Nestin, Lama2*, and *Cd248* (Cho et al., 2003; MacFadyen et al., 2005; Murfee et al., 2005; Armulik et al., 2011; Menezes et al., 2014; De La Fuente et al., 2017; Guimarães-Camboa et al., 2017). However, the most commonly employed marker for pericyte populations, *Pdgfr*β, is ubiquitously expressed in cNCCs, and several of these markers are expressed in other cell populations, including vascular smooth muscle and even endothelial cells themselves (Guimarães-Camboa et al., 2017; Stebbins et al., 2019). We show that Shh pathway activation in cNCCs leads to increased expression of several presumptive pericyte markers, including *Cspg4*, *Cd248*, *Lama2*, and *Tbx18* (Figure 5). Determining the fidelity and specificity of these genes as markers of cNCC-derived pericytes is an important area of future investigation that could drive new approaches to examining the role of pericytes in craniofacial morphogenesis, tissue healing, and repair.

The study of cleft lip pathogenesis has historically focused on proliferation and apoptosis during tissue outgrowth and fusion. Here, we provide evidence that microvascular morphogenesis may be an underlying OFC mechanism and warrants further investigation. While Shh signaling is known to be crucial in craniofacial morphogenesis, our findings point to a previously unrecognized role for Shh signaling in pericyte-endothelial cell interaction where perivascular Shh signaling promotes microvascular stability. Disruption of Shh signaling may therefore interfere with pericyte differentiation and function, leading to microvascular dysfunction, a lack of tissue outgrowth, and cleft of the upper lip. These findings support microvascular morphogenesis as a novel focus for the role of Shh in birth defects as well as for understanding the etiology of structural birth defects more generally.

## Data Availability Statement

All datasets presented in this study are included in the article/Supplementary Material.

## Ethics Statement

The animal study was reviewed and approved by the University of Wisconsin–Madison, School of Veterinary Medicine Institutional Care and Use Committee.

## Author Contributions

RL and MRS conceived the studies and wrote the manuscript. MRS, HC, MJS, SP, ES, and RL designed the methodology. MRS, HC, VM, and DF conducted the experiments and acquired and analyzed the data. All authors contributed to the article and approved the submitted version.

## Conflict of Interest

The authors declare that the research was conducted in the absence of any commercial or financial relationships that could be construed as a potential conflict of interest.
