# Supplementary material for: Sonic Hedgehog Signaling in Cranial Neural Crest Cells Regulates Microvascular Morphogenesis in Facial Development
Source: Front Cell Dev Biol. 2020 Oct 7;8:590539. doi: 10.3389/fcell.2020.590539 (PMC7575766; doi:10.3389/fcell.2020.590539)
Supplement: Supplementary file 1 [file Data_Sheet_1.docx]

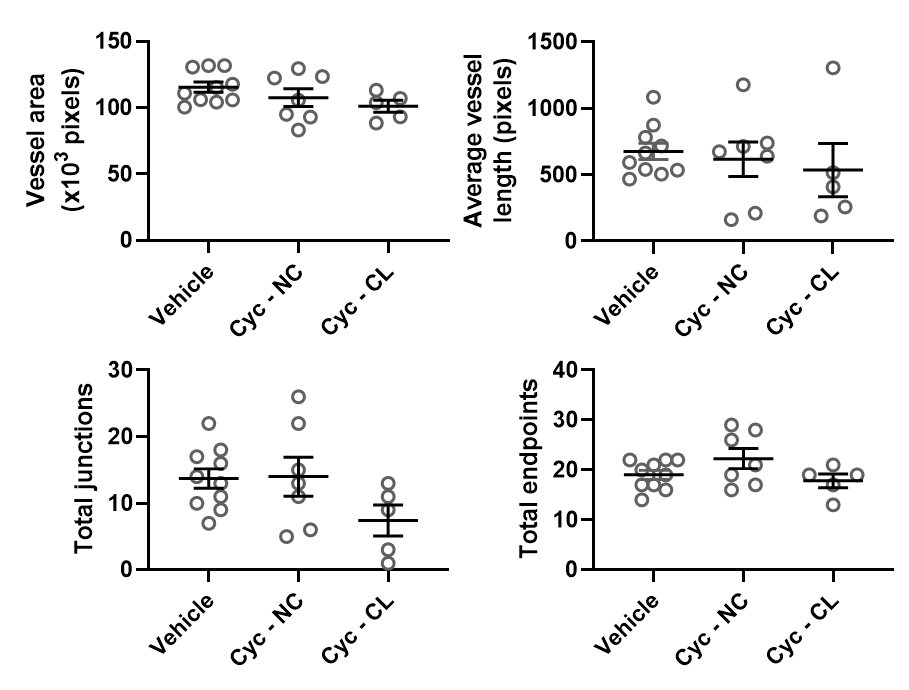


**Supplemental Figure 1.** Microvascular morphogenesis in the proximal MNP. The microvasculature of the proximal medial nasal process from vehicle- and cyclopamine (cyc)-exposed embryos were analyzed using AngioTool. Cyclopamine-exposed embryos were classified as having no cleft (NC) or a cleft lip (CL) phenotype. The vessel area, average vessel lengths, number of junctions, and number of endpoints in the microvasculature from 5-10 embryos per experimental group were analyzed. Measurements for individual embryos are indicated by the icons, and the means ± SEM are shown.


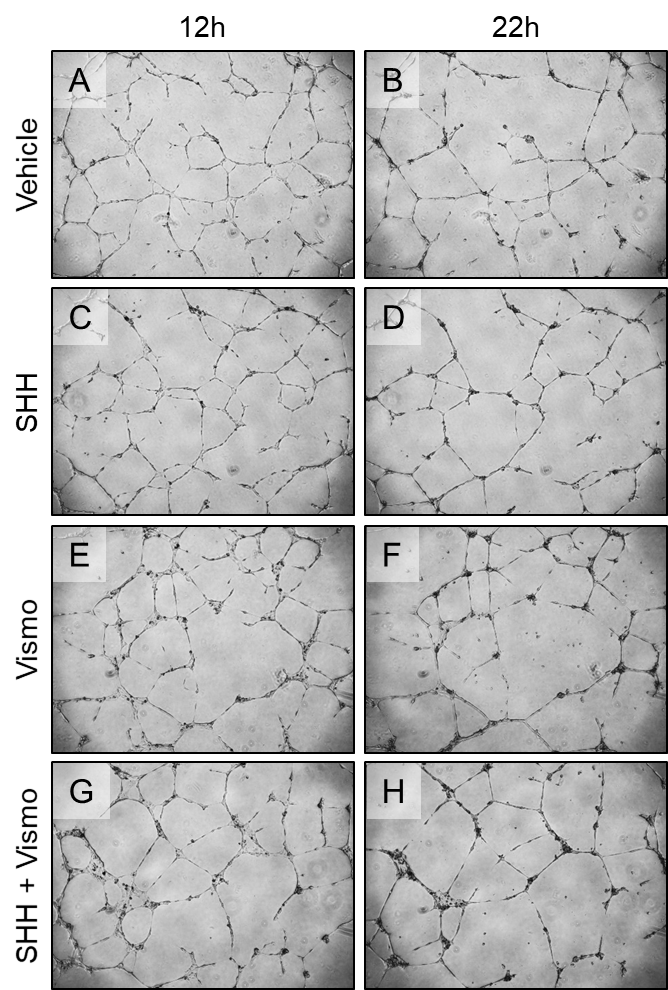


**Supplemental Figure 2.** Phase contrast images for the network schematics shown in Figure 3. **(A-H)** Phase contrast images of HUVECs plated on Matrigel and treated with 0.4 μg/ml SHH and/or 200 nM vismodegib were taken at 12 and 22 hours. Images correspond to the network schematics shown in Figure 3A-H.


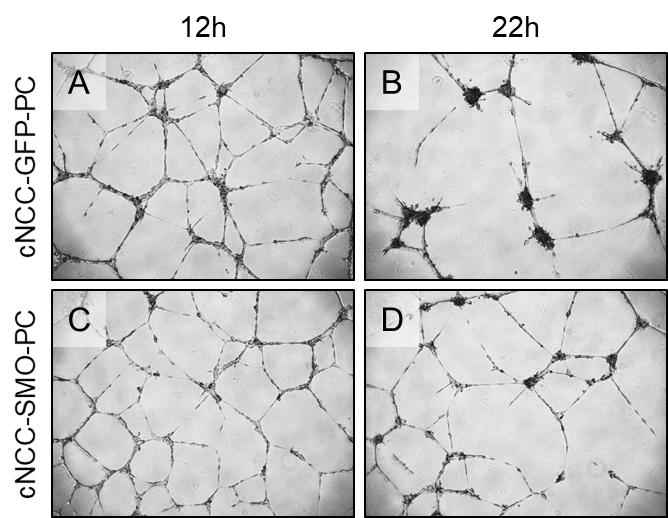


**Supplemental Figure 3.** Phase contrast images for the network schematics shown in Figure 6. **(A-D)** Phase contrast images of HUVECs cocultured with control pericyte-differentiated cNCC (cNCC-GFP-PC) or Shh-activated pericyte-differentiated cNCC (cNCC-SMO-PC) plated on Matrigel were taken at 12 and 22 hours. Images correspond to the network schematics shown in Figure 6A-D.

**
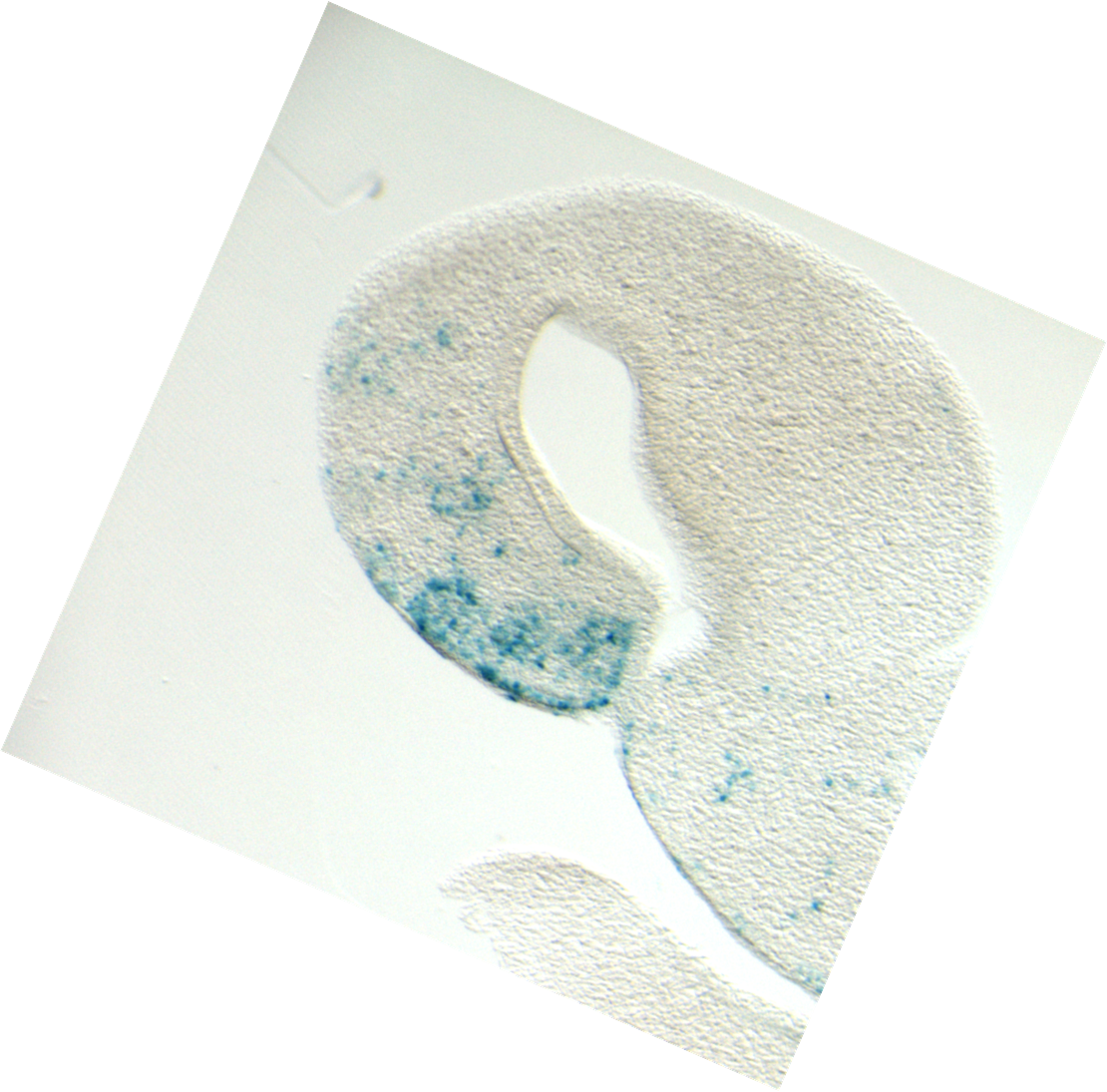
**

**Supplemental Figure 4.** SHH-responsive cells during the critical period of upper lip development specifically localize to the distal tip of the MNP. Timed-pregnant R26R (B6.129S4-*Gt(ROSA)26Sor*^tm1Sor^/J) female mice that were mated to *Gli1-CreER^T2^* (STOCK *Gli1^tm3(cre/ERT2)Alj^*/J) male mice were administered a single dose of 25 mg/kg tamoxifen in corn oil at GD8.75 via intraperitoneal injection. Embryos were harvested at GD11.25, fixed in 4% PFA, and sectioned on a vibrating microtome. Detection of β-galactosidase activity using Xgal was performed to trace the lineage of cells that expressed *Gli1* at GD8.75 during the critical period of upper lip development.
